# Epidemiological Investigation of Osteoarthritis in Middle-Aged Mongolian and Senior Residents of the Inner Mongolia Autonomous Region

**DOI:** 10.5812/ircmj.8303

**Published:** 2013-10-05

**Authors:** Yuewen Wang, Rui Peng, Ruilian Ma

**Affiliations:** 1 Department of Orthopaedics, Affiliated Hospital of Inner Mongolia Medical University, Hohhot, China; 2 Department of Pharmacy, Affiliated Hospital of Inner Mongolia Medical University, Hohhot, China

**Keywords:** Osteoarthritis, Physical Examination, Hypertension

## Abstract

**Background:**

To investigate the prevalence and characteristics of osteoarthritis (OA) in Mongolian middle-aged and senior residents of the Inner Mongolia autonomous region, compared with the prevalence of OA in different regions, to understand the OA-associated factors and provide theoretical evidences for intervention and prevention.

**Objectives:**

Thereby the prevalence, distribution characteristics and correlative factors of OA in Mongolian middle-aged and senior residents in the Inner Mongolia autonomous region were investigated in this study.

**Materials and Methods:**

Rural and urban residents in Hohhot, Baotou and Erdos were selected using stratified, multi-stage and cluster random sampling. 2000 residents aging over 45 filled out questionnaires, underwent specialized physical and X-ray examinations. The factors affecting OA were analyzed by the multivariate unconditional logistic regression.

**Results:**

Obtained total of 1877 questionnaires were completed. 93% of the residents were unaware of OA-related issues. The prevalence of radiological OA and clinical OA (neck OA: 36.72%; waist OA: 44.02%; knee OA: 12.43%; hand OA: 6.83%) were 62.17% and 56.15%, respectively. Urban residents were more subjected to cervical spine (χ^2^ = 8.92, P = 0.005) and less to lumbar spine disease (χ^2^ = 10.32, P = 0.004) compared to rural ones. The prevalence of OA in knees and hands of females (χ^2^ = 8.65, P = 0.003) was significantly higher than males (χ^2^=4.37, P=0.042). The prevalence of OA in postmenopausal females was slightly higher than males (χ^2^ = 3.86, P = 0.052), with no statically significant difference. The risks of OA obviously increased with age. The residents with hypertension, diabetes and atherosclerosis were more subjected to OA.

**Conclusions:**

The prevalence of OA in Mongolian middle-aged and senior residents in part of the Inner Mongolia autonomous region was similar to the other ethnic groups. The prevalence of OA was affected by age, gender, location, hypertension, diabetes, atherosclerosis and osteoporosis. The residents need further educations about OA prevention and treatment.

## 1. Background

Osteoarthritis (OA) has been characterized as a slowly evolving degenerative disease affecting cartilage tissues and bones (1, 2). OA is a common disease of elderly people with higher incidence among females than males. Knee, hip, spines and distal interphalangeal joints that are responsible for human movements are more easily threatened by OA.

The primary and secondary OAs are existing. The former occurs in uncertain manner (patients > 50 years old), in patients without any previous trauma, infection, history of congenital malformation, genetic defect, systemic metabolism or endocrine disorder. The secondary OA occurs based on the original local lesions, such as congenital malformations (e.g. congenital dislocation of hip joints), traumas (e.g. intra-articular fracture), acquired uneven joint surfaces (e.g. ischemic necrosis of bones), unstable joints (e.g. loose joint capsules or ligaments), and bad closure of joint surfaces due to joint deformation (e.g. bandy leg, baker leg) (3, 4).

Preliminary investigations showed that the overall prevalence of OA is approximately 15% (~40 years old: 10 - 17%; > 60 years old: 50%; > 75 years old: 80%), and 53% of OA patients eventually become disabled. Medical issues and expenses for OA are sharply increasing, making the OA prevention and treatment a predominant public health problem (5, 6).

## 2. Objectives

Thereby the prevalence, distribution characteristics and correlative factors of OA in Mongolian middle-aged and senior residents in the Inner Mongolia autonomous region were investigated in this study.

## 3. Patients and Methods

### 3.1. Study Participants

The study protocol of the Inner Mongolia OA study was approved by the Ethic Committee of the Affiliated Hospital of Inner Mongolia Medical University, and informed consents in written form were obtained from all of the participants. The information, including name, gender, age, and address, was obtained from local mayor’s office. The inclusion criterion for this study was 40 years of age or older. Aiming to reflect the targeted population parameters, the Inner Mongolia OA was carried out with reference to the 2010 Chinese population census (7) in three regions of the Inner Mongolia, including Greater Huhhot, Greater Baotou and Greater Erdos, respectively. For each region, the study was conducted in both urban and rural areas given that both areas differed markedly in terms of educational level, household income, mobility and access to the healthcare services.

### 3.2. Methods

#### 3.2.1. Sampling

A classified, multi-stage and cluster random sampling method was utilized. Classification: 1. non-agricultural urban labors in accordance with the survey requirements; 2. agricultural rural labors in accordance with the survey requirements. For stages 1 and 2, purposive sampling was carried out (principle: an area with middle level of economy and education, balanced occupational composition, fair medical conditions and good adaptability to epidemiological investigation and physical examination). Accordingly, one city and one suburb of Hohhot, Baotou and Erdos were selected from January to June 2011, in which the urban and rural areas were further investigated. in stage 3, Urban (3) and rural (2) residents' committees were randomly sampled.

### 3.3. Intervention and Control of OA

OA intervention and control questionnaires were used (8). In the first stage, indoor questionnaires were conducted by investigators (general status, history of present and previous diseases, physical examinations). In the second stage, the cervical spines, lumbar spines, front and lateral knees and front hands of the residents were photographed by X-ray, which were then filled in the questionnaires according to Kellgren & Lawrence grading. In the last stage the finally the OA was diagnosed (9, 10).

### 3.4. Diagnosis Criteria

The following criteria were used in this study:

1) Kellgren & Lawrence OA grading: Grade 0 (without osteophyte), Grade I (slight osteophyte), Grade II (obvious osteophyte without involving joint spaces), Grade III (moderate stenosis of intervertebral space) and Grade IV (obvious stenosis of intervertebral space, accompanied by subchondral osteosclerosis).

 2) Clinical criteria for the final diagnosis of primary OA: The patients who had clinical symptoms and Kellgren & Lawrence grading not lower than Grade II, were excluded from secondary OA based on the most severe segments and parts (11, 12).

### 3.5. Statistical Analyses

Statistical analyses were conducted using a commercial statistical software SPSS for Windows version 15.0 (SPSS, Chicago, IL). Data were presented as mean or percentile. Binary logistic regression analysis was conducted to investigate the associations of the dependent variable presence of osteoarthritis with categorical and/or continuous variables including age (divided into four age groups), gender, body mass index (BMI), area, and conditions of hypertension, atherosclerosis, diabetes, and osteoporosis. Furthermore, the presence of OA in neck, waist, knees, and hands was investigated using binary logistic regression respectively if OA was clinically confirmed. Two-sided P < 0.05 considered as significant. Confidence Intervals of 95% were also provided if applicable.

## 4. Results

In this study, 2000 residents aging with the average age of 58 years old (45 - 92 years old), were randomly sampled out of 1877 complete records (responding rate: 93.85%). The cervical spines, lumbar spines, front and lateral knees and front hands of the grouped residents were photographed by X-ray. Most of (93%) the surveyed residents were unaware about the OA. The population composition and OA prevalence were similar in the three regions.

### 4.1. OA Prevalence of the Residents

Radiological OA: 1167 residents (62.17%); clinical OA: 1054 residents (56.17%), including 36.72% neck OA (387 out of 1054), 44.02% waist OA (464 out of 1054), 12.43% knee OA (129 out of 1054), 6.83% hand OA (72 out of 1054); 50.85% urban residents (536 out of 1054), 49.15% rural residents (518 out of 1054); 46.39% male residents (489 out of 1054), 53.61% female residents (565 out of 1054).

### 4.2. OA Epidemiological Characteristics 

1) Age distribution: The prevalence of OA increased with age, and more parts of the body were affected as well (Spearman correlation coefficient r = 0.271, P < 0.05) (Table 1). 

**Table 1.** Prevalence of OA in Different Age Groups

Age(y)	45 - 54	55 - 64	65 - 74	≥ 75	χ^2^	P
**Overall**	111 (10.56)	291 (27.63)	383 (36.32)	269 (25.49)	48.52	< 0.001
**Neck**	48 (4.56)	118 (11.15)	174 (16.54)	71 (6.76)	15.16	< 0.001
**Waist**	41 (3.88)	100 (9.46)	119 (11.33)	106 (10.10)	28.36	< 0.001
**Knee**	14 (1.37)	52 (4.91)	54 (5.10)	56 (5.28)	26.98	< 0.001
**Hand**	8 (0.75)	22 (2.11)	35 (3.35)	36 (3.39)	6.57	< 0.005

2) Distribution Location x: Urban residents were subjected more to cercival spine (χ ^2 ^= 8.92, P = 0.005) and less to lumbar spine disease (χ ^2 ^= 10.32, P = 0.004) compared to rural ones (Table 2). 

**Table 2.** The Correlation of OA Prevalence and Location Controlling for OA Position

Position	Urban		Rural		χ^2^	P
	Case number	Prevalence, %	Case number	Prevalence, %		
**Overall**	536	50.85	518	49.15	1.21	0.416
**Neck**	225	21.34	162	15.37	8.92	0.005
**Waist**	207	19.64	257	24.38	10.32	0.004
**Knee**	69	6.55	62	5.88	1.66	0.384
**Hand**	35	3.32	37	3.51	0.84	0.623

3) Gender distribution: The prevalence of OA in the knees and hands of females (χ^2 ^= 8.65, P = 0.003) was significantly higher than males (χ^2 ^= 4.37, P = 0.042). The prevalence of postmenopausal females was slightly higher than males (χ^2 ^= 3.86, P = 0.052) with no significant difference (Table 3). 

**Table 3.** Prevalence of OA in Different Gender Groups

Position	Urban		Rural		χ^2^	P
	Case number	Prevalence %	Case number	Prevalence %		
**Overall**	489	46.39	565	53.61	3.86	0.052
**Neck**	215	20.40	213	20.21	1.22	0.314
**Waist**	214	20.30	221	20.97	1.34	0.256
**Knee**	41	3.89	89	8.44	8.65	0.003
**Hand**	19	1.80	42	3.98	4.37	0.042

Age, gender, location, body mass index, hypertension, osteoporosis, diabetes and atherosclerosis were used as the independent variables, and the possibilities of suffering from OA were used as the dependent variables, which firstly underwent single-factor screening (menopause was also included solely in women groups). Thereafter, the OA relevant factors (P < 0.2) were incorporated into a multiple regression equation (inclusion criteria for variables: P < 0.05; exclusion criteria for variables: P > 0.1) and underwent multivariate logistic regression analysis (13 - 15). The OA in each position was affected by different factors. The elderly and the residents with hypertension, diabetes and atherosclerosis were susceptible to to OA. Cervical spine and lumbar spine OAs were mainly influenced by locations (urban or rural areas), knee and hand OAs were dominated by gender (Table 4). 

**Table 4.** Logistic Regression Analysis Results

Position (Factor)	B	χ^2^	Exp (B) and 95% CI, No.(%)	P
**Neck**				
Hypertension	0.526	6.384	1.723 (0.539 - 4.236)	0.015
Atherosclerosis	0.877	16.984	2.525 (1.165 - 7.231)	0
Diabetes	0.612	6.048	1.905 (0.237 - 6.014)	0.014
Osteoporosis	0.475	7.269	1.741 (0.339 - 5.475)	0.007
Age, y	0.262	11.546	1.458 (0.259 - 3.216)	0
Location	0.741	18.344	2.102 (0.231 - 7.421)	0
**Waist**				
Hypertension	0.653	11.624	1.964 (0.418 - 5.335)	0.003
Atherosclerosis	0.425	4.685	1.631 (0.079 - 5.746)	0.041
Diabetes	1.203	22.345	2.985 (0.114 - 11.269)	0
Osteoporosis	0.589	10.036	1.849 (0.151 - 3.657)	0.003
Age	0.527	14.104	1.724 (0.419-7.227)	0
Gender	0.674	7.526	2.033 (1.009 - 6.542)	0.006
**Knee**				
Hypertension	0.592	5.847	1.846 (0.275 - 8.426)	0.022
Diabetes	0.776	7.698	2.247 (0.746 - 12.370)	0.008
Osteoporosis	0.694	8.236	2.014 (0.497 - 3.628)	0.005
Age	0.725	13.415	2.167 (0.631 - 5.567)	0
**Hand**				
Gender	0.981	6.048	2.548 (0.635 - 6.774)	0.024
Diabetes	1.212	6.826	2.711 (0.397 - 8.904)	0.013

## 5. Discussion

Currently, large-scale epidemiological investigations on OA are still uncommon in most of the countries, all over the world. Peking Hospital reported that the overall prevalence of OA (cervical spine, lumbar spine, knees and hands) nationwide was 46.3%, which increased with age (16). The prevalence of OA in the investigated areas of the Inner Mongolia autonomous region was 56.17%.

Large differences in OA prevalence between countries may be associated with the populations, methods, and diagnosis criteria, etc. Therefore, it is necessary to select similar populations, adopt uniform methods and standardize analyses. This study was carried out by stratified, multi-stage and cluster random sampling. Large-scale or long-term prospective and retrospective studies on OA prevalence and features have been successfully conducted in several developed countries using cohort, control of frequency-matching methods, and ecological researches (17, 18). However, OA-relating issues in China were mainly solved by local hospitals because of the large population, territory and insufficient detection systems. This study did not include the hospital-centered sampling method, which provides evidence for public health units.

Li et al. (19) reported that the elderly residents in six administrative areas, who had high body mass and started drinking alcoholic beverages early were more subjected to the knee OA. Other risk factors, including professional athletes, osteoporosis history, smoking, educational level, gender (being female), having sister or mother with OA, occurred in different regions. Considering that 50% of OA may be inherited, this study lacks the family genetic spectrum analysis (20).

Statistically, diseases such as hypertension and diabetes are positively correlated with OA significantly. Nevertheless, their clinical correlations have never been demonstrated (21). Higher incidence of OA in hypertension and diabetes patients may be resulted to the lesions of the terminal arthroidal cartilage and subchondral bone due to poor blood supply. It has been previously reported that hyperglycemia is associated with the high incidence and severity of OA, and hypercholesteremia is associated with systemic OA, independently (22). Findlay found that (23) vascular diseases play an important role in OA. Arterial ischemia or venous stasis after vascular lesions may lead to ischemia and hypoxia even metabolic waste accumulation, which induce local inflammation and edema. Thus, ischemia and hypoxia continue to deteriorate. The long bone ends located at the distal blood vessels are more vulnerable to subchondral bone necrosis, micro-fracture, sclerosis, articular cartilage ischemia and hypoxia, and chondrocyte apoptosis, which can induce inflammatory response and accelerate cartilage destruction. It has been clinically verified that OA patients suffer from serious edema (24, 25).

In summary, 93% of the surveyed residents were uninformed about OA prevention and treatment. Therefore, publicizing the OA-relating knowledge and correct exercises are essential. These exercises including:

 1) Promoting the knowledge about OA in communities.

2) Altering the unhealthy daily manners. 

3) Providing daily exercise guidelines for residents, especially the elderly. 

4) Postponing the diseases by correct living manners and exercises.

 5) Treating the patients who have been diagnosed as OA with steroid injection at local joints and orally administered medicine (e.g. anti-inflammatory and detumescent painkiller aspirin, non-steroid anti-inflammatory drugs (26). 

6) Prescribing a novel anti-inflammatory drug (cyclooxygenase-2 inhibitor) that does not trigger the gastric ulcer and gastrorrhagia of OA patients (27).
